# A Chinese Expert Consensus on the Artificial Intelligence Proficiency of Medical Students: Competencies and the Multi‐Modal Assessment

**DOI:** 10.1002/hcs2.70049

**Published:** 2026-02-17

**Authors:** Mengchun Gong, Jiao Li, Yonghui Ma, Bo Jin, Wei Chen, Yan Hou, Li Hong, Tianwen Lai, Bohan Zhang, Ge Wu, Zhirong Zeng

**Affiliations:** ^1^ GMC Lab, School of Biomedical Engineering Guangdong Medical University Dongguan China; ^2^ Digital Health China Technologies Ltd Beijing China; ^3^ Guangzhou Women and Children's Medical Center Guangzhou China; ^4^ Institute of Medical Information Chinese Academy of Medical Sciences & Peking Union Medical College Beijing China; ^5^ School of Medicine Xiamen University Fujian China; ^6^ Big Data Center Guangdong Medical University Dongguan China; ^7^ Department of Nephrology, The First Affiliated Hospital Sun Yat‐sen University Guangzhou China; ^8^ School of Biostatistics Peking University Beijing China; ^9^ Shanghai Children's Medical Center Shanghai Jiao Tong University School of Medicine Shanghai China; ^10^ The First Dongguan Affiliated Hospital Guangdong Medical University Dongguan China

**Keywords:** AI proficiency, artificial intelligence (AI), assessment, competency framework, medical education

## Abstract

**Background:**

Artificial intelligence (AI) is transforming healthcare, demanding reevaluation of medical education. China's “New Medical Education” initiative urgently requires a standardized AI literacy framework for medical students to address fragmented standards, rapid technological evolution, and insufficient localized ethical norms.

**Objective:**

To establish a Chinese expert consensus defining core AI competencies and a multi‐modal assessment framework for medical students.

**Methods:**

A multidisciplinary (including medical education, clinical medicine, medical AI, public health, and medical ethics) expert group (*n* = 32) developed an initial competency list based on the “Knowledge‐Skills‐Attitude” Medical Competency Model. Two Delphi rounds (100% response rate; consensus threshold: mean ≥ 4.0, CV ≤ 0.25) refined the framework. Core competencies were prioritized via Analytic Hierarchy Process (AHP). The final consensus document was established after multiple expert group meetings.

**Results:**

The consensus defines AI literacy for medical students as a comprehensive attribute for integrating AI into professional knowledge, clinical practice, research, and health management. It comprises a 21‐item Competencies of AI Proficiency (CAIP) list across knowledge (eight indicators), skills (seven indicators), and attitude (six indicators) dimensions. Key competencies prioritized include understanding AI's role in multidisciplinary knowledge integration (CAIP3), identifying AI output biases (CAIP4), understanding health data governance (CAIP2), maintaining physician‐led AI‐assisted diagnosis (CAIP16), and identifying AI diagnostic biases (CAIP12). A multi‐modal assessment framework is recommended, including paper‐based/computerized tests for knowledge, situational judgment tests (SJTs) for attitudes, and objective structured clinical examinations (OSCEs) with a specific “AI Clinical Decision Conflict Scoring Scale” for skills. A multi‐stage dynamic assessment system (“Pre‐enrollment–Pre‐clinical–Post‐clinical”) is proposed for longitudinal tracking. Educational integration pathways emphasize embedding AI literacy modularly from early undergraduate years, constructing an integrated curriculum covering fundamental principles, advanced large model applications (e.g., prompt engineering, agent development), and ethical considerations, supported by a “digital twin hospital platform.”

**Conclusion:**

This consensus provides authoritative, China‐specific guidance for defining and assessing medical students' AI literacy, adhering to national policies and regulations. It offers a core action framework for optimizing AI integration into medical education, fostering future healthcare professionals proficient in both AI technology and medical humanism, with a commitment to dynamic updating to adapt to evolving AI advancements.

## Graphical abstract

This study proposes an AI literacy framework for medical students, structured around knowledge, skills, and attitudes with 21 core competencies. It stresses physician accountability, and bias vigilance alongside medical humanism. AUnidentified multimodal assessment system is introduced, using tests, SJTs, and OSCEs, with progresss tracked longitudinally. The framework advocates for localized education integrating regulations, a modular curriculum, digital twin training, and alignment with domestic healthcare needs.

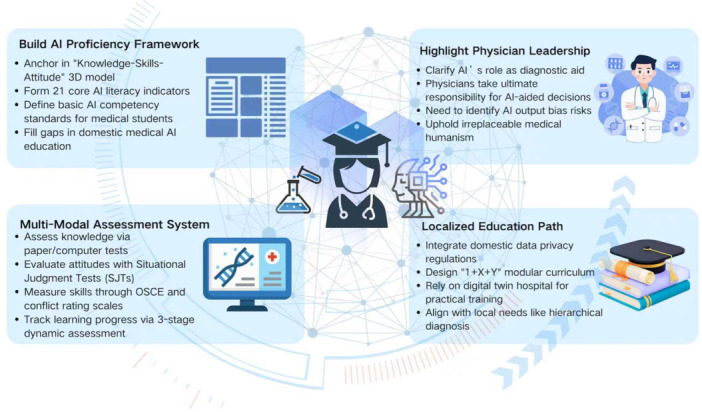

## 1 Background

The profound development of artificial intelligence (AI) technology is driving systemic transformation across all sectors of society, with the healthcare industry undergoing historic restructuring [1]. By enhancing diagnostic and therapeutic precision, optimizing decision‐making efficiency, and reshaping service models, AI continuously unleashes revolutionary potential to improve healthcare quality and patient experience. It has become a core engine for advancing the modernization of healthcare systems and the transformation of medical education paradigms [2]. Currently, AI is deeply integrated throughout the clinical decision support chain, undertaking critical functions such as diagnostic assistance, data interpretation, and workflow automation, triggering structural changes in medical division of labor and professional competencies. AI technology has penetrated beyond a mere tool level into the core of the diagnostic and therapeutic system, driving a shift towards a “human‐AI collaborative decision‐making” mode^l^ [3]. While compensating for physicians' knowledge gaps and workload through automated analysis, it also poses challenges for cultivating medical students' clinical reasoning and defining diagnostic responsibilities [4]. Consequently, medical education is compelled to evolve beyond its traditional boundaries, proactively shaping a new generation of practitioners who are not only clinically competent but also adept at navigating, critiquing, and leading within an AI‐augmented healthcare environment.

During this strategic period where AI empowers high‐quality health development, China's medical education shoulders a dual mission, the accomplishment of which rests fundamentally with medical educators: it must both respond to the disruptive challenges posed by rapid technological iteration on the physician competency matrix and proactively lay the talent foundation for a digital‐intelligent healthcare ecosystem. Against the backdrop of New Medical Education construction and the digital transformation of medical education, AI literacy for medical students has transcended technical operation, evolving into a multidimensional competency system integrating engineering thinking, ethical judgment, and clinical decision‐making [5]. Grounded in the Competency‐Based Medical Education (CBME) theoretical framework [6], future physicians need to develop a multifaceted role identity as “AI Collaborators,” “Ethical Overseers,” and “Data Stewards.” This requires mastering the logic of generative AI in diagnostics and therapeutics, adhering to healthcare data security norms, while steadfastly preserving the irreplaceable value of humanistic care and professional judgment [7]. Currently, Chinese medical education faces practical challenges such as the rapid iteration of AI technical standards, insufficient localization of ethical norms, and the absence of robust assessment systems. There is an urgent need to establish an AI literacy competency framework aligned with the requirements of the “Implementation Plan for the Education Power Promotion Project during the 14th Five‐Year Plan period,” providing guidance for cultivating innovative talents with international perspectives adapted to the smart healthcare ecosystem [8]. Therefore, guided by the national strategies of “Healthy China 2030” and “New Medical Education,” this consensus integrates evidence‐based medical research and international advancements, synthesizing the wisdom of multidisciplinary experts. It aims to establish Chinese‐specific standards for medical students' AI literacy competencies and assessment, providing a core action framework for building a future‐oriented healthcare talent support system.

## 2 Methods

**Figure 1:**
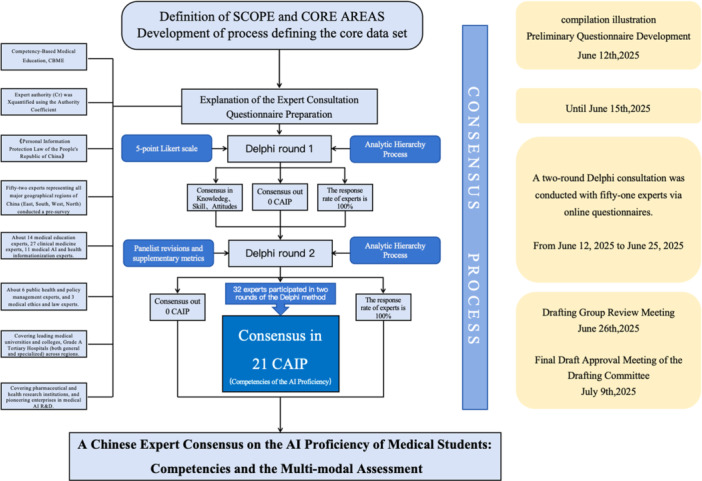
Flow chart of the development and consensus process for the CAIP core data set. CAIP, Competencies of AI Proficiency.

A consensus expert group was responsible for conducting correspondence inquiries, evaluations, proposing revisions, and finalizing the AI literacy competency list and assessment framework. The composition of the expert group adhered to the principle of “multidisciplinary, multi‐regional, integration of medicine, education, research, and industry,” comprising 13 medical education experts, 27 clinical medicine experts, 11 medical AI and health informatics experts, six public health and policy management experts, and three medical ethics and law experts. Participants represented leading medical universities, tertiary Grade A general and specialized hospitals across various regions, medical and health research institutions, and leading medical AI R&D enterprises. A writing group conducted literature reviews and searches, drafted the initial assessment list and framework, organized Delphi expert consultations [9], summarized and analyzed consultation results, and prepared the initial consensus draft. A secretariat handled information collection and expert liaison. The flow chart gives an overview of all steps taken during the project (Figure 1).

### 2.1 Expert Selection Criteria

Experts were selected based on the following criteria: (1) professional title: all experts held senior professional titles (Professor, Chief Physician, or equivalent) in their respective fields; (2) academic background: experts possessed doctoral degrees or equivalent research experience in medicine, biomedical engineering, health informatics, medical education, or related disciplines; (3) AI‐related experience: a minimum of 3 years of practical or research experience in medical AI, health data science, or digital health applications. Experts were drawn from leading medical universities, tertiary Grade A hospitals, research institutions, and medical AI enterprises to ensure comprehensive coverage of clinical, educational, technical, and policy perspectives.

#### 2.1.1 Distribution of Expert Affiliations

The expert panel comprised 51 members from 13 provinces, municipalities, and autonomous regions across China, ensuring broad geographical representation and mitigating regional bias. The institutional distribution was as follows:

Medical Universities and Research Institutes: 28 experts (54.9%), including representatives from Guangdong Medical University, Xiamen University, Peking Union Medical College, and Peking University.

Tertiary Grade A Hospitals: 19 experts (37.3%), including clinicians and administrators from Guangzhou Women and Children's Medical Center, The First Affiliated Hospital of Sun Yat‐sen University, Shanghai Children's Medical Center, and others.

Medical AI Enterprises and R&D Institutions: four experts (7.8%), representing leading companies in health AI and data governance.

This balanced composition ensured that the consensus integrated perspectives from academia, clinical practice, industry, and policy‐making, thereby enhancing its authority and applicability.

### 2.2 Generation of Items Methodology and Procedure

This study followed the theoretical framework of the “Medical Competency Model,” which emphasizes that medical professionals require integrated competencies across three dimensions: knowledge, skills, and attitudes [6]. Referencing competency classifications from international medical education organizations (e.g., Accreditation Council for Graduate Medical Education, ACGME) and domestic counterparts, the knowledge dimension primarily embodies “cognitive competency,” corresponding to the “medical expert” role. It focuses on AI fundamentals, cross‐specialty integration pathways, and risk identification. The skills dimension embodies “functional competency,” corresponding to the “collaborator” and “practitioner” roles, emphasizing AI tool operation, human–AI collaborative decision‐making, and adaptation to clinical contexts. The attitude dimension embodies “social competency,” corresponding to the “professional” and “health advocate” roles, focusing on safety boundaries, ethical responsibilities, social equity, and healthcare accessibility within technology application [10, 11]. High‐risk assessment scenarios evaluate students' adaptability and problem‐solving abilities in specific diagnostic and therapeutic situations, corresponding to “situational competency.” To enhance the scientific rigor and practicality of assessment, multiple evaluation methods were designed. An objective structured clinical examination (OSCE) station proposal, based on the “behavioral anchoring” principle (observable behavior) of the competency model, simulates scenarios like AI misdiagnosis leading to patient disputes to assess skill application. Situational judgment tests (SJTs) measure the attitude dimension, aligning with the “critical incident technique” for assessing professional values [12]. Regarding educational integration and practical orientation, options for optimizing teaching content and curriculum design anchor competency development across three stages: knowledge construction (algorithm principles), skill transformation (AI tool application), and attitude internalization (ethical case studies). Recommendations for clinical learning scenarios are based on promoting “spiral progression of competency” (from simulation to real‐world settings). Furthermore, emphasis was placed on localization and content innovation. For instance, the knowledge dimension incorporates China's “Personal Information Protection Law” to address domestic healthcare data compliance requirements; the “High‐Risk Scenario Assessment” option includes “critical care decision‐making,” aligning with clinical pain points in domestic tertiary hospitals. The attitude dimension addresses “social responsibility of AI technology in bridging healthcare resource disparities (e.g., promoting AI tools in primary care),” reflecting China's hierarchical diagnosis and treatment strategy. Building on this foundation, the initial competency list and assessment framework for medical student AI literacy were constructed by further incorporating practical needs identified in clinical practice.

### 2.3 Consensus Process and Outcome Scoring

#### 2.3.1 Delphi Procedure

This study employed the Delphi method. Following a preliminary survey, 32 experts were invited to participate in two formal consultation rounds. The expert panel was drawn from key universities, tertiary hospitals, research institutions, and leading medical AI development enterprises to ensure a multidisciplinary perspective. Among the panelists, 30 individuals (93.8%) held senior professional titles and 30 (93.8%) held doctoral degrees, indicating profound academic and practical expertise. The response rate for both consultation rounds was 100%, demonstrating high engagement. The expert authority coefficient was 0.79, confirming the high credibility and reliability of the consensus reached. During two rounds of Delphi questionnaire consultation, experts rated the importance (“1” = very unimportant, “5” = very important) of AI literacy competency items and the feasibility (“1” = very infeasible, “5” = very feasible) of the assessment framework using Likert‐5 scales. Inclusion criteria required meeting both “mean ≥ 4.0” and “coefficient of variation < 0.25.”

#### 2.3.2 Priority Setting

The Analytic Hierarchy Process (AHP) determined core competency priorities. The optimal stage for AI literacy education was inquired via single‐choice questions. Multiple‐choice, short‐answer, and practical questions gathered input on “High‐Risk Scenario Assessment,” “Optimization Design of AI Literacy Teaching Content and Curriculum System,” and “Clinical Learning Scenario Suggestions.” Expert feedback was synthesized to form the initial draft consensus on the medical student AI literacy competency list, assessment framework, and educational integration pathways.

The AHP was applied to determine the relative weights and priority ranking of the 21 AI literacy competency indicators. A hierarchical structure was constructed with the goal of “AI literacy competency” at the top, the three dimensions (knowledge, skills, and attitude) as criteria, and the 21 specific competency indicators as alternatives.

#### 2.3.3 Judgment Matrix Construction and Consistency Testing

Expert judgments on the relative importance of elements within each dimension were collected and synthesized to construct pairwise comparison matrices using a 1–9 scale. The consistency of each judgment matrix was rigorously evaluated using the consistency ratio (CR). A CR value less than 0.10 indicates acceptable consistency.

#### 2.3.4 Relative Weight Ranking of Key Indicators

The global weights of all 21 competency indicators, calculated by multiplying the dimension weights by the local weights within each dimension, are presented in descending order in Table S1. This AHP‐based weighting provides a quantitative foundation for prioritizing educational objectives and assessment focus within the AI literacy framework.

#### 2.3.5 Finalization

This draft was further reviewed, discussed, and finalized by a consensus review panel to form the “Expert Consensus on the Competency Framework and Assessment System for AI Literacy in Medical Students.” Following two writing group review meetings (June 26, 2025; July 9, 2025) and one full Expert Group finalization session (July 12, 2025), the full consensus text was established.

## 3 Results

### 3.1 Results of the Consensus Process

In total, 51 experts received a WeChat invitation to participate in the Delphi vote. The response rate was 100% in both rounds. All 21 items met consensus criteria (mean range: 4.25–4.84; SD range: 0.37–0.80).

For the results of the AHP, items of the three dimensions (knowledge, skills, and attitude) demonstrated high consistency of the evaluation; the conformance rate of the first‐level indexes was 0.00; and the conformance rates of the second‐level indexes were 0.02, 0.02, and 0.03, respectively. The detailed pairwise comparison matrices, consistency test results (CR and *λ*_max_), and local weights for all dimensions and indicators are provided in Table S2. In summary, the judgment matrix for the three dimensions (knowledge, skills, and attitude) showed perfect consistency (CR = 0.0000; *λ*_max_ = 3.0000), with equal weights assigned to each dimension (0.3333). The judgment matrix for the eight knowledge indicators (knowledge dimension, CR = 0.0204; *λ*_max_ = 8.2009), eight skills indicators (skills dimension, CR = 0.0239; *λ*_max_ = 8.2362), and five attitude indicators (attitude dimension, CR = 0.0327; *λ*_max_ = 5.1464) demonstrated acceptable consistency (CR = 0.0204; *λ*_max_ = 8.2009). All CR values were below the 0.10 threshold, confirming the logical consistency of expert judgments and the reliability of the derived weights. At the drafting group review meeting, all items were discussed and evaluated with respect to the importance of the item for AI literacy evaluation.

### 3.2 Contents of Medical Student AI Literacy Competency List (Competencies of AI Proficiency [CAIP])

Based on the “Knowledge‐Skills‐Attitude” three‐dimensional competency model, and incorporating literature review and expert experience, the CAIP list for medical students was constructed. It comprises 3 first‐level (dimensions) and 21 second‐level (competency) indicators (Table 1).

**Table 1:** Medical student AI literacy competency list (CAIP).

Dimension	Competency (CAIP)
Knowledge	1. Understand the basic knowledge, application value in healthcare, and limitations of AI.
	2. Master the fundamental theories and technologies of health data governance.
	3. Comprehend the role of AI in integrating multidisciplinary knowledge and addressing overspecialization and knowledge fragmentation.
	4. Identify AI biases stemming from data quality and sources; understand how medical AI may exacerbate health inequities and systemic discrimination in resource allocation.
	5. Be aware of AI‐related healthcare regulations (e.g., HIPAA/GDPR/China's Personal Information Protection Law, Interim Measures for the Management of Generative Artificial Intelligence Services) and ethical boundaries.
	6. Understand the transparency limitations inherent in the reasoning logic and decision‐making processes of large AI models.
	7. Recognize the diverse application scenarios of AI in future clinical medicine.
	8. Understand the theoretical impact of AI technology on clinical reasoning training and the potential risk of cognitive substitution.
Skills	9. Operate AI‐assisted diagnostic tools, including but not limited to imaging recognition systems, multi‐disease decision support systems, and generative AI‐assisted medical record drafting systems.
	10. Integrate AI outputs into clinical decision‐making, modifying AI‐suggested plans that conflict with clinical reality.
	11. Objectively reference AI suggestions to formulate personalized diagnosis and treatment plans based on individual patient differences.
	12. Identify AI diagnostic biases in simulated and real clinical scenarios through methods such as data traceability and logical verification.
	13. Utilize AI tools for rapid acquisition of cross‐specialty knowledge.
	14. Master the fundamental techniques and key tools for secure data usage, de‐identification, and protection of personal sensitive information.
	15. Possess the skill to integrate medical AI cases and tools into clinical learning and other training processes.
	16. Be able to explain the function and limitations of AI tools to patients.
Attitude	17. Adhere to the principle of physician leadership, utilizing AI‐assisted diagnosis appropriately.
	18. Physicians bear ultimate responsibility for AI‐assisted decisions.
	19. Prioritize the use of clinically validated AI tools and refrain from using experimental AI products in routine clinical practice.
	20. Pay attention to the social responsibility of AI technology in narrowing healthcare resource disparities, emphasizing the promotion of AI tools in primary care.
	21. Prudently select and reference AI tools based on professional medical judgment.

#### 3.2.1 Knowledge Dimension

This dimension includes eight competency indicators covering core principles, technological awareness, ethical/regulatory knowledge, application scenarios, and limitations.

#### 3.2.2 Skills Dimension

This dimension includes eight competency indicators covering AI tool application, clinical integration and judgment, risk identification and correction, interdisciplinary information integration, and integration of AI tools in medical education and practice.

#### 3.2.3 Attitude Dimension

This dimension includes five competency indicators covering human‐centered philosophy and physician leadership awareness, ethical responsibility and duty to explain, scientific prudence and risk awareness, and the value stance of promoting healthcare equity.

### 3.3 Key AI Literacy Competencies for Priority Focus

Based on AHP calculations of weights for CAIP indicators and thorough expert discussion and deliberation, the top five prioritized competencies based on their global weights were CAIP17 (attitude, weight: 0.1176), CAIP1 (knowledge, weight: 0.0928), CAIP18 (attitude, weight: 0.0891), CAIP12 (skills, weight: 0.0866), and CAIP21 (attitude, weight: 0.0626). The following core competencies are identified as priorities:

#### 3.3.1 CAIP17 (Attitude Dimension)

Clearly recognize the auxiliary nature of AI in the diagnostic process. Acknowledge that all diagnostic and therapeutic decisions should be led by the physician's professional knowledge and clinical judgment. AI tools serve only as decision‐support aids and cannot replace the independent judgment of medical professionals [13].

#### 3.3.2 CAIP1 (Knowledge Dimension)

This competency requires medical students to master AI's core mechanisms and to recognize its clinical value in improving diagnostics and personalized treatment. Crucially, they must understand AI's inherent limitations: heavy reliance on training data (risking bias amplification), lack of explainability (“black box” issue), and absence of clinical context awareness. This foundational knowledge ensures students can utilize AI's benefits while recognizing its boundaries to safeguard clinical decision safety and patient rights.

#### 3.3.3 CAIP18 (Attitude Dimension)

Physicians bear ultimate responsibility for AI‐assisted decisions. Operational details of “physician responsibility.” The physician's duty of professional review of AI‐generated content (e.g., verifying diagnostic basis, excluding algorithmic bias) and the right of final decision signature constitute the basis of responsibility. If medical malpractice occurs due to failure to fulfill the review duty, the physician bears primary responsibility. If errors result from algorithm design flaws or data fabrication, the medical institution and AI developer bear joint liability [13].

#### 3.3.4 CAIP12 (Skill Dimension)

Able to identify potential biases in AI system diagnostic outputs through data provenance tracing, logical verification, and other means in simulated or real clinical scenarios. Possess the ability to reasonably question, verify, and correct AI‐generated content, thereby enhancing the safety and reliability of AI in medical practice.

#### 3.3.5 CAIP21 (Attitude Dimension)

This competency requires medical students to ensure AI always supports rather than replaces professional medical judgment. Students must internalize the principle that clinical judgment remains paramount, while AI serves as a supporting tool requiring careful evaluation based on specific clinical contexts and evidence levels. Given AI's inherent limitations, including data biases, algorithmic “black boxes,” and limited contextual understanding, its outputs may contain errors or outliers. Students must therefore critically evaluate AI‐generated content using clinical experience and evidence‐based knowledge, actively identifying and correcting potential risks while accepting full responsibility for final decisions.

## 4 Discussion

### 4.1 Positioning Within the Global Context of Medical AI Education

The urgent need to integrate AI into medical education is a globally recognized challenge. International scholars have consistently argued that AI is not merely a new tool but a transformative force that necessitates a fundamental rethinking of physician competencies [4, 8]. Previous research has effectively outlined the high‐level domains required for AI literacy, such as foundational knowledge, clinical application, and ethical reasoning [8, 14]. Wartman and Combs, for instance, compellingly called for a “reimagining” of medical curricula to prepare future physicians for a symbiotic relationship with AI [14]. Similarly, frameworks from influential bodies like the ACGME and CanMEDS provide a robust, role‐based structure for competency development in general terms [10, 11].

However, a gap persists between these foundational principles and their practical implementation in specific national contexts. As Masters noted, the rapid iteration of AI technology often outpaces the adaptation of educational frameworks, leading to a “fragmented” approach in many institutions [4]. This consensus directly addresses this implementation gap by moving from the “what” to the “how.” While prior research has successfully established the agenda for change [8, 14], this study provides a methodologically rigorous and locally contextualized blueprint for achieving it. Our work complements international calls to action by delivering a granular, consensus‐based competency list (CAIP) and a multi‐modal assessment system, thereby operationalizing high‐level principles into measurable educational outcomes.

### 4.2 Requirements for Medical Student Training in the Era of Generative Medical AI

To adapt to the rapidly evolving demands of the AI era, medical student training must clarify the following orientations and requirements, including “Confident Collaboration With AI,” “Identifying and Mitigating AI‐Related Risks,” “Clarifying Legal and Ethical Responsibility Boundaries,” and “Ensuring Effective Patient Privacy Protection.”

The competencies outlined in our CAIP framework respond directly to the challenges identified in prior literature. The imperative for “Confident Collaboration With AI” aligns with Rampton et al.'s prediction that future physicians must function as “information integrators” who can critically evaluate AI‐generated insights within complex clinical contexts [8]. Our framework provides a specific set of skills, from tool operations to output integration, required to achieve this role.

Similarly, the focus on “Identifying and Mitigating AI‐Related Risks” addresses the well‐documented ethical limitations of algorithmic fairness and the “black box” problem in healthcare machine learning [9]. McCradden et al. highlighted these risks, and our consensus translates their ethical concerns into teachable and assessable competencies, such as identifying biases (CAIP12) and maintaining physician oversight (CAIP17, CAIP18) [13].

Furthermore, the requirements for “Clarifying Legal and Ethical Responsibility Boundaries” and “Ensuring Effective Patient Privacy Protection” are grounded in the growing body of literature on AI governance. Our integration of regulations like China's “Personal Information Protection Law” and “Interim Measures for the Management of Generative AI Services” provides a localized response to the global need for legal and ethical frameworks in AI education, as discussed in studies on data privacy in Chinese healthcare settings [15, 16].

### 4.3 Recommendations for Assessment Framework Design

#### 4.3.1 Knowledge Assessment

Utilize paper‐based or computerized tests to evaluate medical students' understanding of AI fundamentals, technical principles, risk management, ethical regulations, application scenarios, and limitations. Construct a test question bank (≥ 50 items), primarily using single‐choice questions, adhering to the principles of “comprehensive assessment” and “moderate difficulty.”

#### 4.3.2 Attitude Assessment

SJTs are applied by presenting typical scenarios related to AI technology assisting clinical diagnosis and treatment work (such as human–machine decision conflict, patients questioning AI‐assisted diagnosis, and so on) and multiple possible behavioral responses of the test‐taker in that situation. The test‐taker is required to make selections and evaluations based on prompts, thereby objectively scoring attitudes.

#### 4.3.3 Skills Assessment

Adopt an OSCE format. Design a series of simulated AI‐assisted clinical work scenarios (e.g., AI misdiagnosis causing patient dispute, conflict between generative AI notes and clinical reality). Utilize models, standardized patients, or real patients. Configure the “AI Clinical Decision Conflict Scoring Scale” (Table 2) to quantitatively assess AI literacy across knowledge, professional attitudes, and skills in three dimensions: Data Verification Ability (20 points), Ethical Judgment (30 points), and Doctor–Patient Communication (50 points).

**Table 2:** AI clinical decision conflict OSCE scoring scale.

Assessment dimension	Scoring criteria
Data validation ability	Ability to identify source bias/sample limitations in AI output data.
Ethical judgment	Correctly cites the “Personal Information Protection Law” when handling patient data.
Clinician–patient communication	Ability to explain the limitations of AI suggestions in plain language.

#### 4.3.4 High‐Risk Scenario Assessment

It is recommended to prioritize the inclusion of the following typical scenarios highly associated with AI, possessing clinical complexity or ethical risks, for practical or contextualized assessment to enhance the relevance and effectiveness of the assessment: (1) Multi‐system disease diagnosis: assess students' competence in conducting health assessments, diagnoses, and outcome predictions for multi‐system diseases based on AI‐generated content. (2) Critical care decision support: examine students' use of AI decision support systems in the auxiliary diagnosis and treatment process for critical and emergent conditions. (3) Rare disease identification: evaluate students' ability to use AI tools to expand diagnostic thinking and identify uncommon diseases. (4) Patient data breach response: strengthen students' ability to identify and manage security and privacy risks associated with AI systems.

#### 4.3.5 Multi‐Stage Dynamic Assessment

To construct a closed‐loop “Basic‐Clinical‐Professional” AI literacy competency cultivation system, it is recommended to establish a multi‐stage assessment system (“Pre‐enrollment–Pre‐clinical–Post‐clinical”) to dynamically track the development of students' AI literacy competency across different learning stages. This includes:

1. Initial Assessment (Semester 1): administer a 30‐item AI fundamentals questionnaire to understand the baseline cognition of AI principles among medical students, providing a basis for stratified teaching.
2. Pre‐clinical Assessment (after completing university medical courses, before clinical internship): aims to assess the mastery level of AI foundational knowledge and technical application skills before entering clinical practice.
3. Post‐clinical assessment (after clinical internship): aims to assess students; comprehensive ability and professional demeanor in understanding, evaluating, and applying generative AI content within real‐world healthcare settings.

### 4.4 Synthesis With International Frameworks and Innovations of This Consensus

The development of this consensus occurred against the backdrop of a global exploration of AI competencies in medical education. Internationally, several pioneering frameworks have been proposed, such as those from the AAMC and AMA, which effectively outline high‐level, principle‐based competencies across broad domains like “Fundamental Knowledge,” “Clinical Application,” and “Ethics” [8, 14]. These frameworks provide an essential foundational agenda for change, highlighting the transformative role of AI and the need for a “reimagining” of medical education. However, they often exhibit limitations for direct implementation: (1) lack granular, actionable competency indicators tailored to specific clinical workflows and decision‐making conflicts; (2) they are less integrated with a comprehensive “Knowledge‐Skills‐Attitude” competency model, potentially leaving behavioral and attitudinal competencies underdefined; (3) proposed assessment methods that are often not as multimodal and specific; (4) they may not be fully contextualized to the specific legal, ethical, and healthcare system contexts of non‐Western countries like China [4, 8].

This Chinese expert consensus addresses these gaps and introduces several key innovations and points of differentiation, providing a methodologically rigorous and locally contextualized blueprint for implementation:

1. A Comprehensive and Structurally‐Rigorous Competency Framework with Quantitative Prioritization: Grounded explicitly in the well‐established “Knowledge‐Skills‐Attitude” model [5], this consensus provides a structured, granular, and measurable list of 21 competencies (CAIP). This moves beyond principles to directly link AI literacy to core physician roles (e.g., medical expert, professional) [11]. A significant advance is the use of the AHP to establish a quantitative priority ranking of these competencies. The top‐ranked competencies, “physician‐led diagnosis” (CAIP17) and “ultimate physician responsibility” (C2/CAIP18), give concrete, weighted form to ethical warnings about algorithmic fairness and the irreplaceable value of human judgment [13], translating critical ethical concerns into core, prioritized educational objectives.
2. An Integrated Multi‐modal Assessment System Bridging the Theory‐Practice Gap: While written tests for knowledge are standard, this consensus specifies a tailored, multi‐modal system. The use of SJTs for attitudes is supported by evidence of their validity in assessing professional attributes [12]. More innovatively, the OSCE station with a bespoke “AI Clinical Decision Conflict Scoring Scale” represents a concrete response to the CBME call for assessments that evaluate competency integration in realistic scenarios [6]. It provides a practical tool for assessing the “situational competency” essential for safe practice in AI‐augmented environments, a challenge not fully addressed by existing international guidelines.
3. Deep Localization and Practical Implementation Pathways: This consensus is deeply contextualized for China. It incorporates local regulations (e.g., Personal Information Protection Law) into competencies and addresses domestic clinical priorities like critical care decision‐making. The proposed “1 + X + Y” curriculum model (1 core AI literacy course establishes foundations; X specialized literacy courses, such as AI+Medical Imaging, integrate technical skills with disciplines; Y cutting‐edge projects connect to senior‐year innovation practice) offers a structured pathway to achieve the “spiral progression” of competency advocated in CBME literature [6]. The concept of a “digital twin hospital platform” provides a forward‐looking, risk‐free environment for practical training, operationalizing the call for students to learn in “real‐world healthcare settings.”

The proposed “1 + X + Y” model is being piloted through a structured approach: the core course (“1”) establishes foundational knowledge, as demonstrated in “Fundamentals of AI in Medicine” at institutions like Guangdong Medical University; modular courses (“X”) integrate AI into disciplines like medical imaging within existing curricula; and advanced innovation (“Y”) is realized through practicums like prompt engineering workshops. Supporting this, the development of the “digital twin hospital platform” follows a phased roadmap: from creating high‐risk OSCE scenarios (Phase 1), to expanding virtual clinical workflows for data governance practice (Phase 2), and ultimately establishing a full ecosystem for education and AI validation (Phase 3). This integrated strategy provides a clear, actionable pathway for systematically transforming the consensus framework into tangible educational experiences.

## 5 Conclusion

This consensus addresses the epochal demand for AI technology to reshape medical education. Anchored in the “Medical Student Competency Model” as its core framework, it closely aligns with the practical requirements for AI literacy within clinical contexts while strictly adhering to Chinese policies and regulations, such as the “Interim Measures for the Management of Generative Artificial Intelligence Services,” ensuring AI application in medical education complies with data regulations, privacy protection, and ethical norms. This consensus aims to provide authoritative guidance for scientifically assessing medical students' AI literacy, optimizing the integration pathways of AI into medical education, and fostering the innovative development of AI technology in the healthcare domain. As AI technology rapidly evolves, the connotation of medical student AI literacy will continue to deepen, and the models for integrating AI into medical education will diversify. Therefore, this consensus will adhere to a dynamic update principle, continuously optimizing to provide forward‐looking reference for theoretical research and practical exploration of AI‐empowered medical education. This consensus upholds fundamental principles of medical ethics and technological ethics, prohibiting any attempts to completely replace the physician–patient relationship with AI. The ultimate goal of AI literacy education is not to cultivate technology dependents, but to shape guardians who can harness technology while defending the humanistic spirit of medicine.

## Author Contributions

**Mengchun Gong:** conceptualization, writing – original draft, writing – review and editing, project administration, supervision, funding acquisition. **Jiao Li:** investigation, methodology, writing – original drxaft, writing – review and editing, formal analysis. **Yonghui Ma:** project administration, investigation, validation, writing – review and editing. **Bo Jin:** project administration, supervision, investigation, writing – review and editing. **Wei Chen:** validation, writing – review and editing. **Yan Hou:** writing – review and editing, validation. **Li Hong:** writing – review and editing, validation. **Tianwen Lai:** investigation, writing – review and editing. **Bohan Zhang:** writing – review and editing, writing – original draft, visualization, data curation. **Ge Wu:** writing – review and editing, writing – original draft. **Zhirong Zeng:** writing – review and editing, writing – original draft, conceptualization, funding acquisition, project administration, supervision, methodology.

## Ethics Statement

The authors have nothing to report.

## Consent

The authors have nothing to report.

## Conflicts of Interest

Mengchun Gong is a staff member of Digital Health China Technologies Ltd., Beijing, China. All authors declare no conflicts of interest.

## Supporting information

Supplementary Table S1. Global weights and ranking of the 21 AI literacy competencies for medical students based on the Analytic Hierarchy Process.

Supplementary Table S2. AHP Judgment Matrices, Consistency Test Results, and Local Weights for the AI Literacy Competency Framework.

## Data Availability

The data that support the findings of this study are available from the corresponding author upon reasonable request.
