# Supplementary material for: A Chinese Expert Consensus on the Artificial Intelligence Proficiency of Medical Students: Competencies and the Multi‐Modal Assessment
Source: Health Care Sci. 2026 Feb 17;5(1):49–57. doi: 10.1002/hcs2.70049 (PMC12946706; doi:10.1002/hcs2.70049)
Supplement: Supplementary file 1 — Supplementary Table S1. Global weights and ranking of the 21 AI literacy competencies for medical students based on the Analytic Hierarchy Process. [file HCS2-5-49-s001.docx]

Supplementary Table S1. Global weights and ranking of the 21 AI literacy competencies for medical students based on the Analytic Hierarchy Process.

| Rank | Competency Code | Global Weight | Description (Abbreviated) | Dimension |
| --- | --- | --- | --- | --- |
| 1 | CAIP17 | 0.1176 | AI-assisted diagnosis must be physician-led | Attitude |
| 2 | CAIP1 | 0.0928 | Understand basic AI knowledge, value & limitations | Knowledge |
| 3 | CAIP18 | 0.0891 | Physicians bear ultimate responsibility for AI decisions | Attitude |
| 4 | CAIP12 | 0.0866 | Identify biases in AI diagnostic outputs | Skills |
| 5 | CAIP21 | 0.0626 | AI application based on professional judgment | Attitude |
| 6 | CAIP5 | 0.0582 | Know AI-related regulations & ethical boundaries | Knowledge |
| 7 | CAIP10 | 0.0538 | Integrate AI output into clinical decisions | Skills |
| 8 | CAIP11 | 0.0538 | Objectively reference AI advice based on patient context | Skills |
| 9 | CAIP7 | 0.0475 | Recognize AI's application scenarios in future clinics | Knowledge |
| 10 | CAIP9 | 0.0388 | Operate AI-assisted diagnostic tools | Skills |
| 11 | CAIP8 | 0.0387 | Understand AI's impact on clinical thinking | Knowledge |
| 12 | CAIP19 | 0.0375 | Prioritize clinically validated AI tools | Attitude |
| 13 | CAIP14 | 0.0298 | Use AI tools to acquire cross-specialty knowledge | Skills |
| 14 | CAIP15 | 0.0298 | Understand data security & de-identification techniques | Skills |
| 15 | CAIP3 | 0.0266 | Understand AI's role in integrating multidisciplinary knowledge | Knowledge |
| 16 | CAIP2 | 0.0266 | Master theories of health data governance | Knowledge |
| 17 | CAIP4 | 0.0266 | Identify bias from data quality/source | Knowledge |
| 18 | CAIP20 | 0.0265 | Promote AI tools to reduce inequality in resource-poor areas | Attitude |
| 19 | CAIP16 | 0.0234 | Integrate AI tools in clinical learning | Skills |
| 20 | CAIP13 | 0.0175 | Communicate AI's role & limits to patients | Skills |
| 21 | CAIP6 | 0.0164 | Understand transparency limits in AI decision-making | Knowledge |

*The competencies are ranked by their global weight, which represents their relative priority within the overall AI literacy framework. The weights sum to 1.0000.
