# Supplementary material for: A Chinese Expert Consensus on the Artificial Intelligence Proficiency of Medical Students: Competencies and the Multi‐Modal Assessment
Source: Health Care Sci. 2026 Feb 17;5(1):49–57. doi: 10.1002/hcs2.70049 (PMC12946706; doi:10.1002/hcs2.70049)
Supplement: Supplementary file 2 — Supplementary Table S2. AHP Judgment Matrices, Consistency Test Results, and Local Weights for the AI Literacy Competency Framework. [file HCS2-5-49-s002.docx]

Supplementary Table S2. AHP Judgment Matrices, Consistency Test Results, and Local Weights for the AI Literacy Competency Framework.

Part A: Goal Level - AI Literacy Competency Dimensions

| AI Literacy Competency | Knowledge | Skills | Attitude | Weights (Wi) |
| --- | --- | --- | --- | --- |
| Knowledge | 1 | 1 | 1 | 0.3333 |
| Skills | 1 | 1 | 1 | 0.3333 |
| Attitude | 1 | 1 | 1 | 0.3333 |
| Consistency Ratio (CR): 0.0000; λmax: 3.0000 | | | | |

Part B: Knowledge Dimension (CR: 0.0204; λmax: 8.2009)

| Knowledge | CAIP1 | CAIP2 | CAIP3 | CAIP4 | CAIP5 | CAIP6 | CAIP7 | CAIP8 | Local Weights (Wi) |
| --- | --- | --- | --- | --- | --- | --- | --- | --- | --- |
| CAIP1 | 1 | 3 | 3 | 3 | 2 | 4 | 3 | 3 | 0.2783 |
| CAIP2 | 0.333 | 1 | 1 | 1 | 0.5 | 2 | 0.5 | 0.5 | 0.0798 |
| CAIP3 | 0.333 | 1 | 1 | 1 | 0.5 | 2 | 0.5 | 0.5 | 0.0798 |
| CAIP4 | 0.333 | 1 | 1 | 1 | 0.5 | 2 | 0.5 | 0.5 | 0.0798 |
| CAIP5 | 0.5 | 2 | 2 | 2 | 1 | 3 | 2 | 2 | 0.1745 |
| CAIP6 | 0.25 | 0.5 | 0.5 | 0.5 | 0.333 | 1 | 0.333 | 0.5 | 0.0491 |
| CAIP7 | 0.333 | 2 | 2 | 2 | 0.5 | 3 | 1 | 2 | 0.1426 |
| CAIP8 | 0.333 | 2 | 2 | 2 | 0.5 | 2 | 0.5 | 1 | 0.1161 |

Part C: Skills Dimension (CR: 0.0239; λmax: 8.2362)

| Skills | CAIP9 | CAIP10 | CAIP11 | CAIP12 | CAIP13 | CAIP14 | CAIP15 | CAIP16 | Local Weights (Wi) |
| --- | --- | --- | --- | --- | --- | --- | --- | --- | --- |
| CAIP9 | 1 | 0.5 | 0.5 | 0.333 | 2 | 2 | 2 | 2 | 0.1164 |
| CAIP10 | 2 | 1 | 1 | 0.5 | 3 | 2 | 2 | 2 | 0.1613 |
| CAIP11 | 2 | 1 | 1 | 0.5 | 3 | 2 | 2 | 2 | 0.1613 |
| CAIP12 | 3 | 2 | 2 | 1 | 3 | 3 | 3 | 3 | 0.2597 |
| CAIP13 | 0.5 | 0.333 | 0.333 | 0.333 | 1 | 0.5 | 0.5 | 0.5 | 0.0524 |
| CAIP14 | 0.5 | 0.5 | 0.5 | 0.333 | 2 | 1 | 1 | 2 | 0.0893 |
| CAIP15 | 0.5 | 0.5 | 0.5 | 0.333 | 2 | 1 | 1 | 2 | 0.0893 |
| CAIP16 | 0.5 | 0.5 | 0.5 | 0.333 | 2 | 0.5 | 0.5 | 1 | 0.0703 |

Part D: Attitude Dimension (CR: 0.0327; λmax: 5.1464)

| Attitude | CAIP17 | CAIP18 | CAIP19 | CAIP20 | CAIP21 | Local Weights (Wi) |
| --- | --- | --- | --- | --- | --- | --- |
| CAIP17 | 1 | 2 | 3 | 3 | 2 | 0.3527 |
| CAIP18 | 0.5 | 1 | 3 | 3 | 2 | 0.2672 |
| CAIP19 | 0.333 | 0.333 | 1 | 2 | 0.5 | 0.1125 |
| CAIP20 | 0.333 | 0.333 | 0.5 | 1 | 0.333 | 0.0796 |
| CAIP21 | 0.5 | 0.5 | 2 | 3 | 1 | 0.1879 |
